# The influence of connective tissue grafting on the reconstruction of a missing facial bone wall using immediate implant placement and simultaneous bone reconstruction: a retrospective long-term cohort study

**DOI:** 10.1186/s40729-024-00533-2

**Published:** 2024-05-18

**Authors:** Andreas Kuebler, Robert Noelken

**Affiliations:** 1 Private Practice for Oral Surgery, Paradiesplatz 7-13, 88131 Lindau/Lake Constance, Germany; 2 Department of Oral and Maxillofacial Surgery, University Medical Center, Johannes Gutenberg University of Mainz, Mainz, Germany

**Keywords:** Immediate implants, Bone grafting, Soft tissue grafting, Bone regeneration, Autogenous bone graft, Bone defects, Flapless implant surgery

## Abstract

**Purpose:**

This retrospective cohort study evaluates the influence of connective tissue grafts (CTG) on bone regeneration at implant sites with total loss of the buccal bone wall treated with flapless immediate implant placement (IIP) and reconstruction with autogenous bone chips (AB) within a follow-up of up to 13 years.

**Methods:**

Sixty implants were inserted in 55 patients in sites with total loss of the buccal bone wall between 2008 and 2021. The implants were inserted and the buccal gaps were grafted by AB. A subgroup of 34 sites was grafted additionally with CTG using tunnel technique. Primary outcome was the vertical bone regeneration in height and thickness. Secondary outcome parameters were interproximal marginal bone level, recession, soft tissue esthetics (PES), width of keratinized mucosa (KMW) and probing depths (PPD).

**Results:**

Mean follow-up period was 60.8 months. In 55 sites a complete vertical bone regeneration was documented. The mean buccal bone level increased by 10.6 mm significantly. The thickness of the buccal bone wall ranged between 1.7 and 1.9 mm, and was significantly thicker in sites without CTG. Interproximal marginal bone level was at implant shoulder level. The mean recession improved significantly by 1.2 mm. In sites with CTG, recessions and PES improved significantly more.

**Conclusions:**

Additional CTG in extraction sites with total buccal bone loss followed by IIP with simultaneous AB grafting led to improved PES and recession, but also to a thinner buccal bone wall compared to sites grafted just with AB.

**Graphical Abstract:**

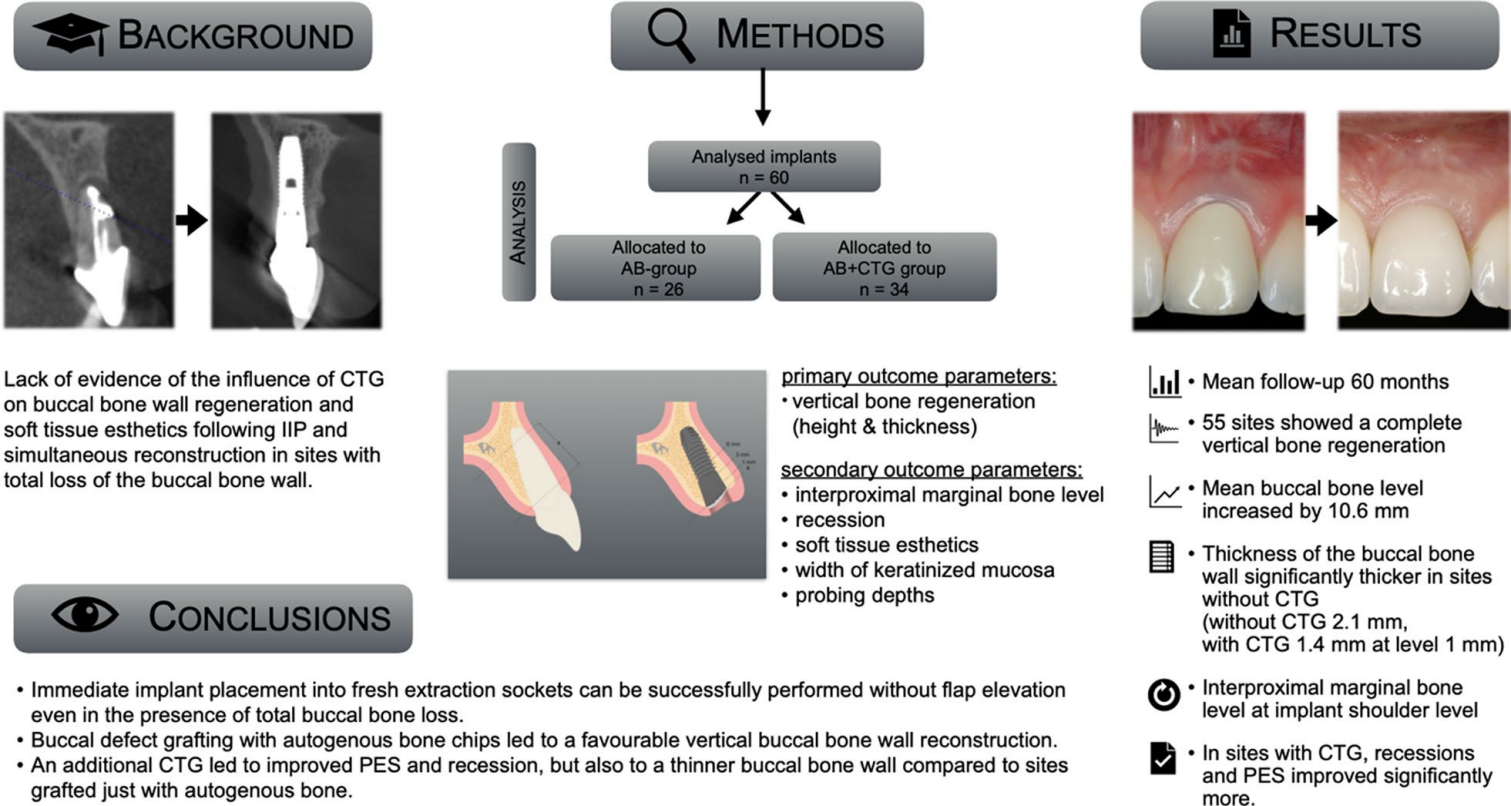

## Background

Apart from implant survival, modern implant dentistry focuses more and more on the reduction of treatment time and preservation of peri-implant bone and soft tissues structures to maintain or rebuild a natural and esthetic emergence profile of the implant supported rehabilitation [16, 28]. The ultimate goal of today´s clinicians is to make implant restorations indistinguishable from natural teeth [7]. The first study describing IIP was published in 1966 by Weiss [53], on IIP in the esthetic zone by Schulte [44], and the concept of IIP and immediate provisionalization has now been established for over 20 years ago [54]. This concept was continually improved over the last 2 decades and many clinical studies showed good esthetic results [10, 12, 17, 32, 34–36, 38].

Based on the results of different studies treatment steps for IIP and immediate provisionalization were definded as follows: atraumatic extraction and flapless techniques [5, 36, 40], palatal/lingual positioning of the implant [20], augmentation of the gap between implant surface and remaining buccal bone wall [29, 33, 42], and immediate provisionalization to support and stabilize the peri-implant emergence profile [18, 19, 36, 49].

In esthetic demanding sites an additional soft tissue augmentation with a connective tissue graft (CTG) is recommended to improve the peri-implant esthetics and to reduce mucogingival recessions [11, 24, 32, 34, 35, 41, 45, 51].

Based on results of former studies in animals and humans concluding that the placement of implants into extraction sites cannot maintain and support the alveolar structures and therefore lead to remodeling and resorption processes especially of the buccal bundle bone [2, 4], the indications for IIP are still restrictive [7, 9]. Therefore IIP is only recommended in sites with a favourable thick buccal bone wall, a thick mucosal biotype and no gingival recession [7, 50, 52].

Unfortunately, the reasons for tooth extraction such as endodontic failure, trauma, advanced periodontal disease and vertical root fracture are mostly associated with a severe alveolar bone resorption, especially of the buccal bundle bone [37, 42]. Chen and Darby stated that every second upper incisor showed buccal bone deficiencies [8]. Thus the reconstruction of the lost structures as a goal of IIP should be considered.

In the last two decades a few retro- and prospective studies in animal [43] and humans were able to demonstrate that a reconstruction of a missing buccal bone wall simultaneously to IIP was possible with autogenous bone (AB) [32–35, 42] and/or bone graft materials (BGM) [14, 47]. A recently published pilot study reported that a missing alveolar buccal bone appears not to be a contraindication for IIP in the esthetic zone if the baseline esthetic situation is accepted by the patient since, with or without BGM grafting, the esthetic situation could not be improved significantly [39].

Even though the positive impact of an additional CTG on the esthetic appearance seems to be obvious, the existing evidence of the influence of a CTG on the reconstruction of the buccal bone wall is very limited and inconsistent.

In a former study of Noelken et al. [32, 34, 35] using IIP and reconstruction of preexisting recessions by augmentation with AB and with or without CTG, a thicker buccal bone wall, and more vertical buccal bone regeneration was observed in sites with CTG. Another study of the same study group observing the impact of implant angulation, soft tissue grafting, and orofacial implant positioning on the buccal bone thickness (BBT) did not report any significant difference in sites using IIP with or without CTG [32, 34, 35]. In contrast, a recently published RCT from Zuiderveld et al. [56] concluded that an additional CTG in sites using IIP and provisionalization was accompanied with more loss of BBT.

The aim of this retrospective long-term cohort study was to evaluate the influence of CTG on the regeneration of the buccal bone wall in height and thickness, as well as on soft tissue esthetics following IIP and simultaneous reconstruction with AB in sites with a total loss of the buccal bone wall after a follow-up period of 1 to 13 years.

## Materials and methods

### Patients

This retrospective cohort study included patients who were in need of a single-tooth implant-supported restoration in anterior or premolar region in the upper or lower jaw. All patients were treated in the period from 06/2008 to 06/2021 in the Private Clinic for Oral Surgery of Prof. Dr. Robert Noelken, Lindau, Germany.

Inclusion criteria were as follows:

- total loss of the buccal bone wall
- IIP of Astra OsseoSpeed implants
- anterior or premolar region in the upper or lower jaw
- flapless procedure
- grafting of the buccal gap with autogenous bone chips
- follow-up period of at least 12 months
- pre- and at least 12 months post-op CB-CT examination.

Exclusion criteria were:

- previous radiation therapy
- systemic bone diseases
- permanent immunosuppressive medication.

Smoking and preexisting periodontal disease were not regarded as exclusion criteria.

**Fig. 1:**
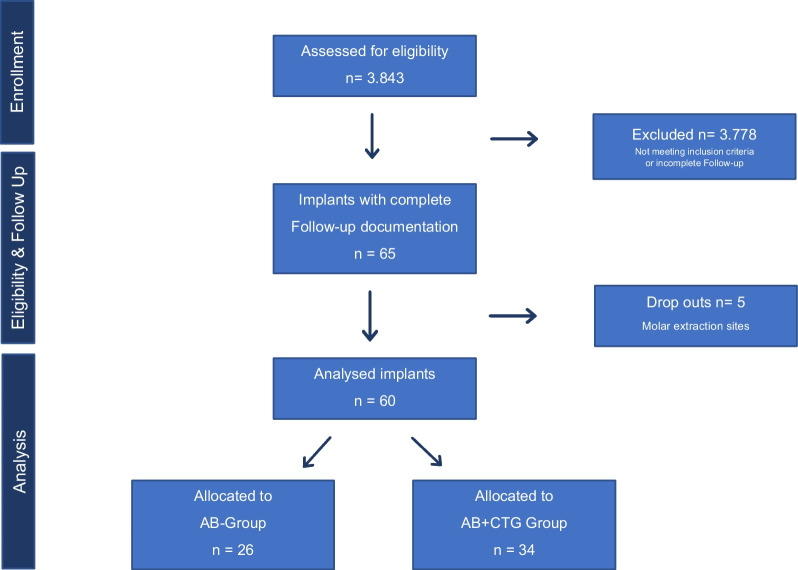
STROBE flow chart of the study population

The preoperative data of 3843 implants were analyzed and the presence of a buccal bone wall was evaluated. In a total of 65 implants a complete loss of the buccal bone wall was documented. 5 implants were placed in a molar extraction site. Sixty implants in 55 patients fulfilled the inclusion criteria with a complete follow-up evaluation of the clinical and radiological status (Fig. 1).

### Ethical approval

Since no study-related additional radiographs or examinations were performed and the publication of the obtained data was analyzed and presented anonymously, the Ethics Committee of the state Bavaria, Germany (file 2023-1005) decided that no consent was necessary for this retrospective cohort study. The study was conducted according to the recommendations of good clinical practice in accordance with the World Medical Association (WMA) Declaration of Helsinki (1975), as revised in 2013 [55].

### Pre-treatment examination

At pre-treatment examination a CB-CT was recorded in all patients to evaluate the dimensions of the alveolar bone before IIP. In most cases intraoral photographs were taken for baseline evaluation of the soft tissue esthetics. The gingival biotype was determined visually by using a periodontal probe according to De Rouck et al. [18, 19].

### Surgical technique

A flapless approach was used for all implants. After a minimally invasive extraction of the condemned teeth by using the periotome technique or Bennex extractor and careful curettage of the alveolar socket under magnification (loops or chairside microscope), the implant sites were prepared according to the manufacturers` instructions. Only Astra Tech implants with OsseoSpeed implant surface were used. The implants were precisely placed in contact to the lingual/palatal bone wall. Simultaneous and flapless bone grafting of the buccal defect between implant surface and buccal soft tissues was performed using autogenous bone chips. Since the implants were inserted without raising a flap to maintain blood supply, a second surgical site at the mandibular ramus was opened to harvest autogenous cortical bone chips by a micro-scraper (Micross, Geistlich, Wolhusen, Switzerland).

Sites with severe recessions, thin biotype, high esthetic expectations or high smile line were grafted additionally with CTG according to the tunnel technique described by Allen [1].

### Immediate and final restorations

The temporary restorations, which were screw-retained and fabricated by a laboratory technician using temporary titanium abutments, were inserted on the day of the implant placement and splinted to the adjacent teeth or implants for at least 8 weeks.

The final restorations were delivered after a minimum of 3 months.

### Follow-up and definition of outcome variables

All patients were examined clinically and radiographically at the time of implant placement and at least 12 months after implant placement.

### Primary outcome parameters

**Fig. 2:**
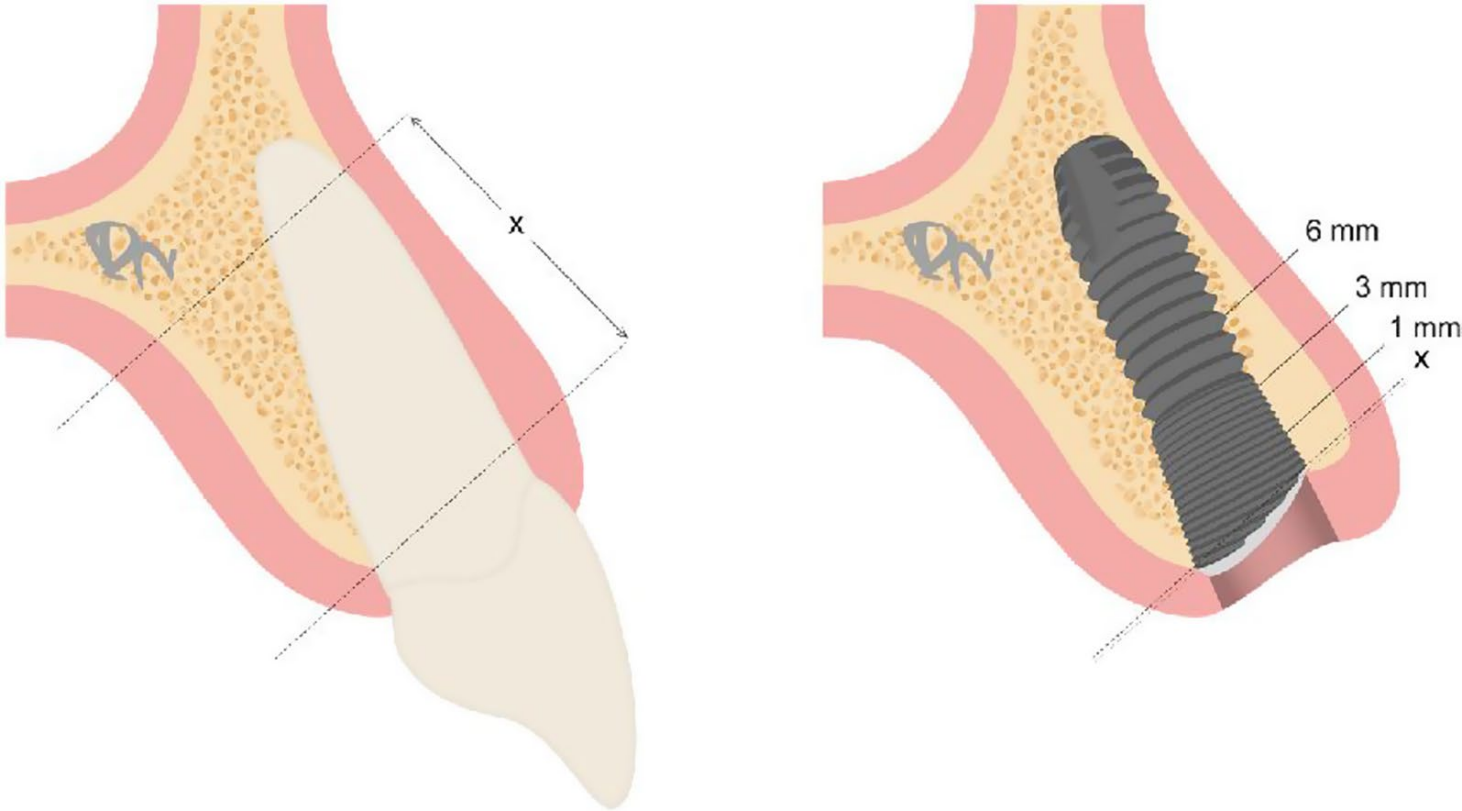
Method of measurement of the depth of preoperative vertical bone loss and the buccal bone wall level in relation to reference level and the buccal bone wall thickness at 1, 3, and 6 mm below reference level at final examination

The primary outcome parameter of this study was the buccal bone wall regeneration in height and thickness. The vertical and horizontal dimension of the buccal bone was evaluated by CB-CT data, specifically by the reconstruction according to the long-axis of the implants. The vertical distance was either measured from 1 mm below the CEJ (cementoenamel junction) to facial bone level (preoperative) or from the first micro-thread (reference level) at the implant sites to the buccal bone level. The thickness of the buccal bone was measured [6, 25] at 1 mm, 3 mm and 6 mm apical to reference level (Fig. 2).

### Evaluation of secondary outcome parameters

#### Interproximal marginal bone level

The interproximal marginal bone height was evaluated by using digital periapical radiographs with paralleling technique. The vertical distances between the level of the mesial and distal bone and the first micro-thread of the implant were measured and designated as positive values and vice versa.

#### Soft tissue recession

The gingival/mucosal recession was calculated in relation to a tangent between the cemento-enamel junctions of the adjacent teeth by a periodontal probe (1 mm calibration, Hu-Friedy Colorvue plastic probe UNC12 SE). In sites where the vertical position of the CEJ has changed over the years, this was observed and calculated in the documentation of the recession measurement.

#### Peri-implant soft tissue esthetics

The esthetics of the per-implant soft tissues was evaluated according to the PES established by Fürhauser [23] prior to surgery and at the time of final follow-up.

#### Peri-implant probing depths

The PPDs were measured at 6 sites around the implant by a periodontal probe with 1 mm calibration.

#### Width of keratinized mucosa

The width of the keratinized and attached mucosa (KMW) was measured at the midbuccal aspect of the implant site by a periodontal probe with 1 mm calibration.

### Statistical analysis

The analysis exploring the linkage between gain in buccal bone height and thickness and the KMW at final examination utilized the Spearman’s rank-based correlations. Subpopulations within the study group (smokers vs. non-smokers, thin vs. thick mucosal biotype, with or without CTG) were compared using the non-parametric Mann–Whitney U-test, since the tested data did not reveal a normal distribution according to the Kolmogorov–Smirnov test. The reported p-values are two-sided. Results were considered statistically significant at p < 0.05. For graphic description, boxplots are given. All calculations were carried out using SPSS 25 (SPSS Inc., Chicago, USA).

All statistical correlation analyses were performed on a “per patient” basis. In case of more than one implant per patient (50 patients received 1 implant; 5 patients received 2 implants), the implant site with the most severe initial buccal bone loss was selected.

## Results

### Study population

The average age of this population (37 women, 18 men) was 52.9 ± 15.9 years (range, 19 to 94 years). Forty-eight were nonsmokers, 7 smokers (5 moderate smokers with 1 to 10 cigarettes a day, and 2 heavy smokers with more than 20 cigarettes). Nineteen patients showed a thin and 36 showed a thick gingival biotype. The implants were inserted to replace central incisors (n = 27), lateral incisors (n = 10), canines (n = 6), premolars (n = 12) in the maxilla, as well as central incisors (n = 2) and premolars (n = 3) in the mandible.

In a subgroup of 34 implants an additional CTG was used to graft sites with soft tissue deficiencies. In 26 implant sites no additional CTG was used. An AstraTech OsseoSpeed implant with a sloped implant shoulder (OsseoSpeed Profile) was used in 29 sites while in 31 sites the same implant but with a flat shoulder was inserted.

### Primary outcomes

#### Patient follow-up

**Fig. 3:**
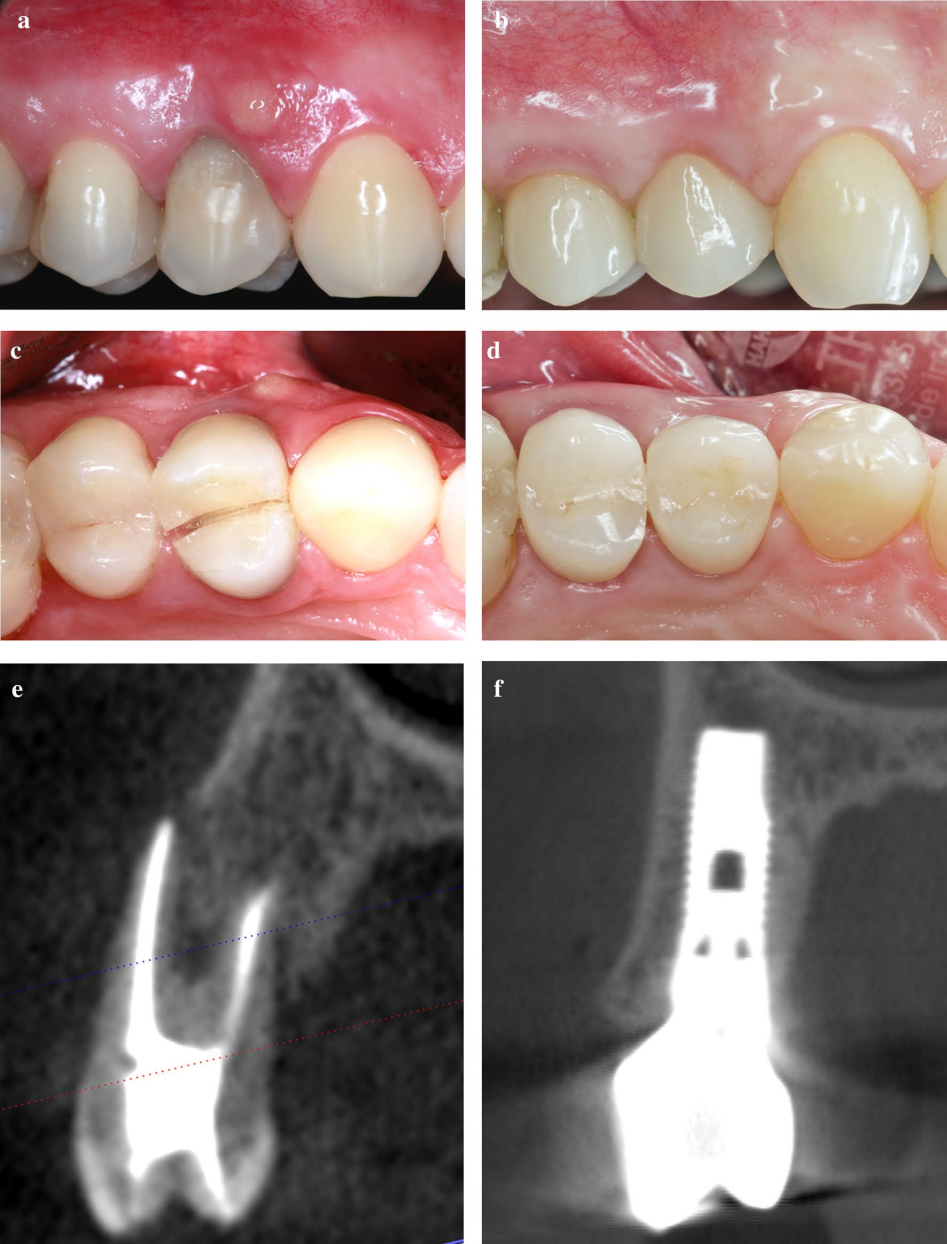
Immediate implant placement in presence of total buccal bone loss and flapless grafting with autogenous bone chips. **a** Initial clinical situation showing a tooth discoloration after root canal treatment, a slight gingival recession and buccal fistula followed to a crown and root fracture. **b** 12 years after IIP and buccal defect grafting with autogenous bone chips a reduction of the mucosal recession was observed. **c** Buccal fistula followed by a crown and root fracture (occlusal view). **d** 12 years after IIP and autogenous bone grafting a slight resorption of the buccal alveolar contour was observed (occlusal view). **e** Preoperative CB-CT showing a chronic interradicular lesion combined with a total loss of the buccal bone wall followed to a crown and root fracture. **f** CB-CT at 12y-follow-up examination shows a complete reconstruction of the buccal bone wall to the level coronal to the implant shoulder

**Fig. 4:**
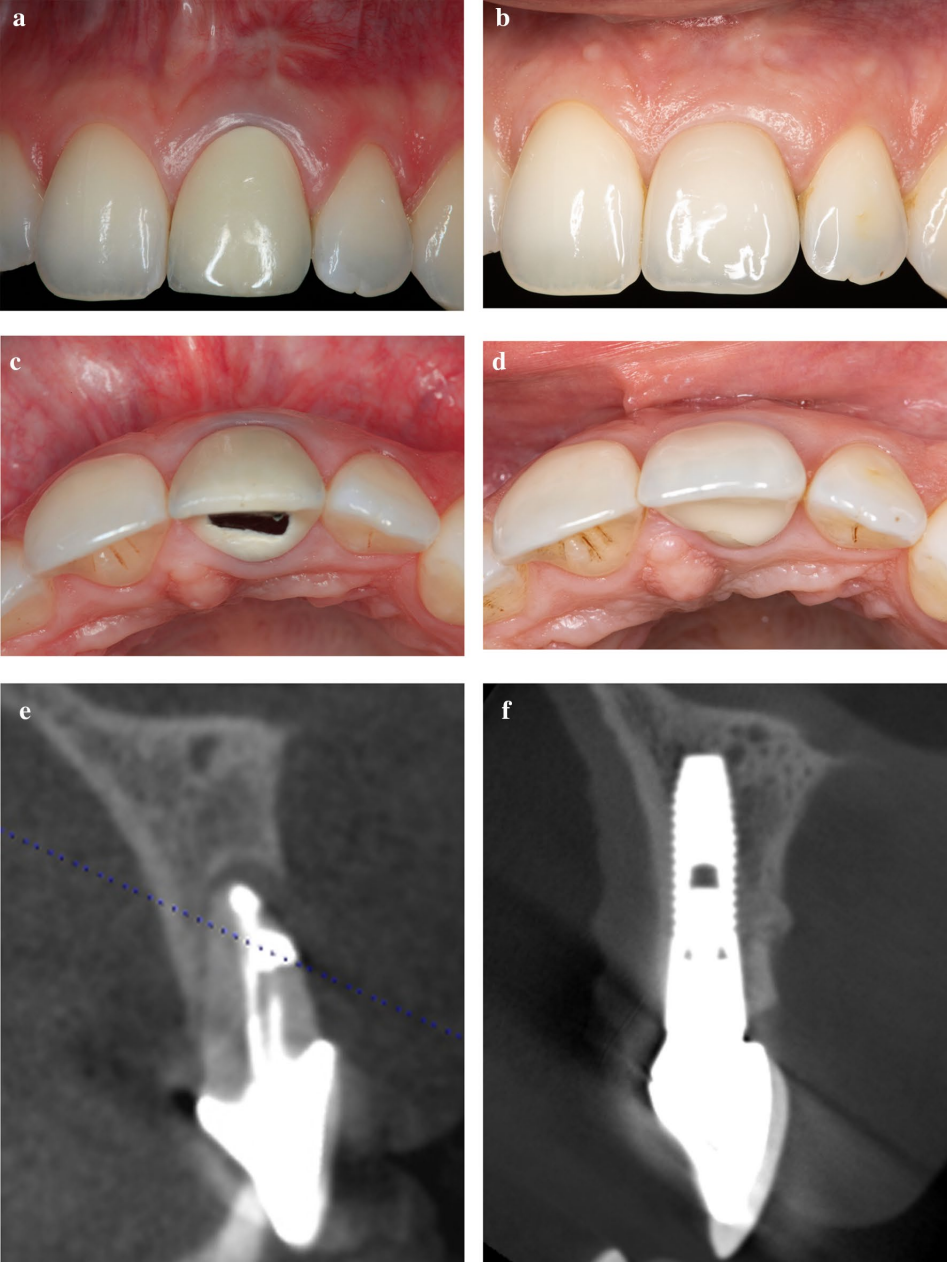
IIP in the presence of total buccal bone loss with flapless grafting with autogenous bone and connective tissue grafting in tunnel technique. **a** Thin mucogingival biotype and loss of buccal bundle bone followed by apicoectomy and vertical root fracture. **b** Healthy and thick peri-implant mucosa and increased soft tissue level 6 years after IIP and facial defect grafting with autogenous bone chips and connective tissue graft. **c** Occlusal view of the initial clinical situation with a thin and natural facial mucosa. **d** Six years after IIP and facial defect grafting with autogenous bone chips and connective tissue graft the facial alveolar contour presents naturally. **e** Preoperative CB-CT showing two root canal treatments, 2 retrograde fillings followed by an apicoectomy and the total loss of the buccal bone wall. **f** A CB-CT at 6y-follow-up examination shows the full reconstruction of the buccal bone wall close to the level of the implant shoulder

Sixty implants in 55 patients were evaluated. Within the mean follow-up period of 60.8 ± 39.3 months (range, 12.6 to 158.9 months) no implant was lost. Representative cases are shown in Figs. 3 and 4.

#### Buccal bone level

**Fig. 5:**
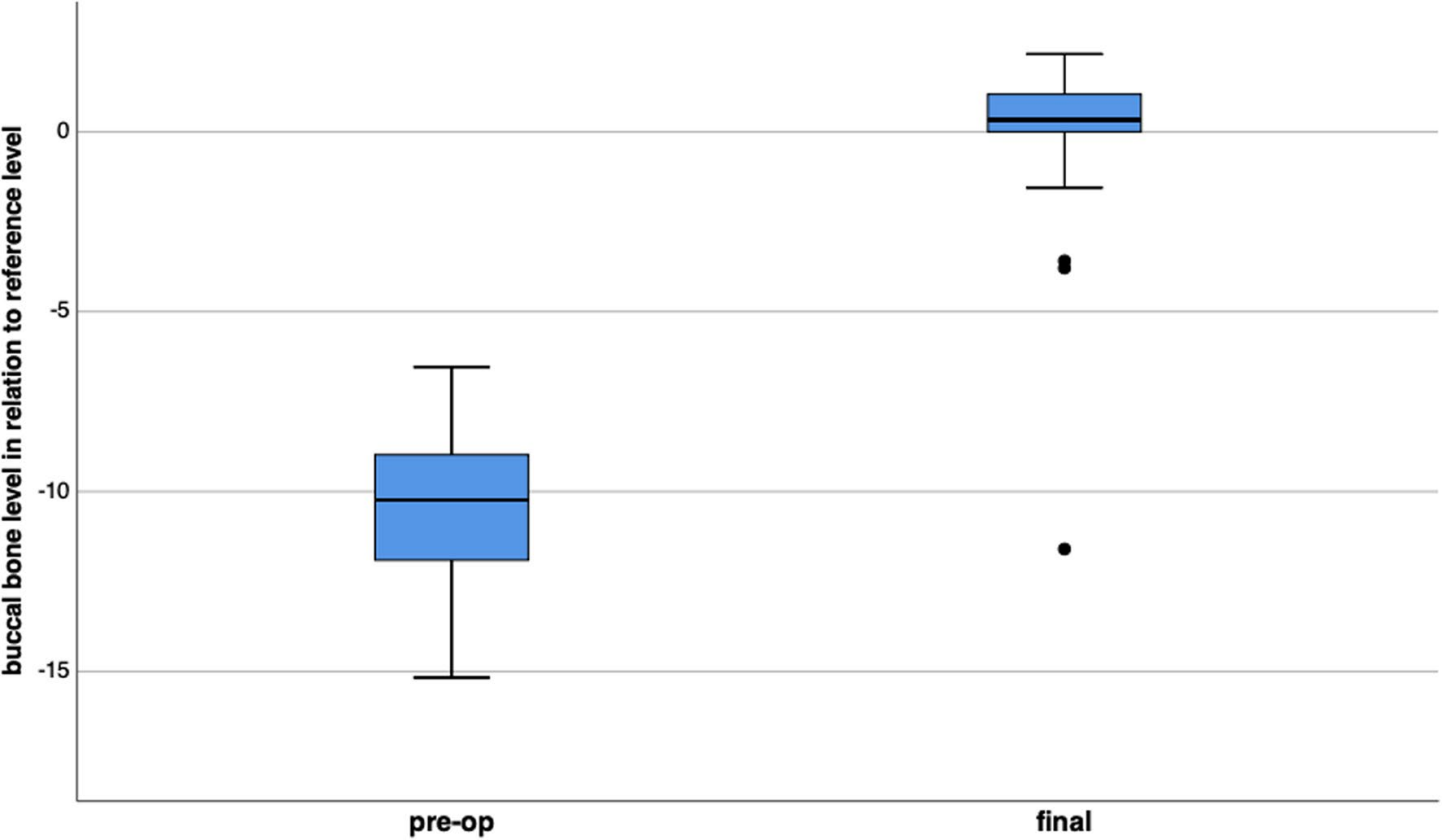
Significant improvement of the buccal bone level from pre-operative to final examination (p < 0.001)

The mean preoperative vertical buccal bone loss was -10.46 ± 2.29 mm. In 55 sites a complete vertical bone regeneration to implant shoulder level was possible. In 4 cases an incomplete bone regeneration and in one case no buccal bone was found radiographically. The mean buccal bone level at implant site was 0.17 ± 1.86 mm coronal to reference level at final examination (Fig. 5). The buccal bone level increased by 10.64 ± 2.93 mm significantly (p < 0.001). The amount of vertical bone regeneration in the subgroup with CTG was 10.88 ± 2.74 mm compared to 10.32 ± 3.18 mm in the subgroup without CTG (p = 0.602). In the subgroup of smokers the vertical bone increase was 9.98 ± 2.14 mm. Non-smokers presented a vertical regeneration of 10.73 ± 3.02 mm. The difference did not reach the level of significance (p = 0.442).

In the subgroup of a sloped implant shoulder configuration, the vertical bone increase was 11.08 ± 2.4 mm. Flat shoulder implants showed a vertical regeneration of 10.22 ± 3.33 mm. The difference did not reach the level of significance (p = 0.280).

#### Buccal bone thickness

Since there was a total loss of the buccal bone wall in all included cases at pre-operative examination with mean depths of 10.46 ± 2.29 mm (range, 6.54 to 15.17 mm), no buccal bone was present at the levels 1, 3, and 6 mm. The mean thickness of the buccal bone wall was 1.73 ± 1.08 mm at level 1 mm, 1.93 ± 1.22 mm at 3 mm and 1.83 ± 1.21 mm at 6 mm at the final follow-up examination.

**Fig. 6:**
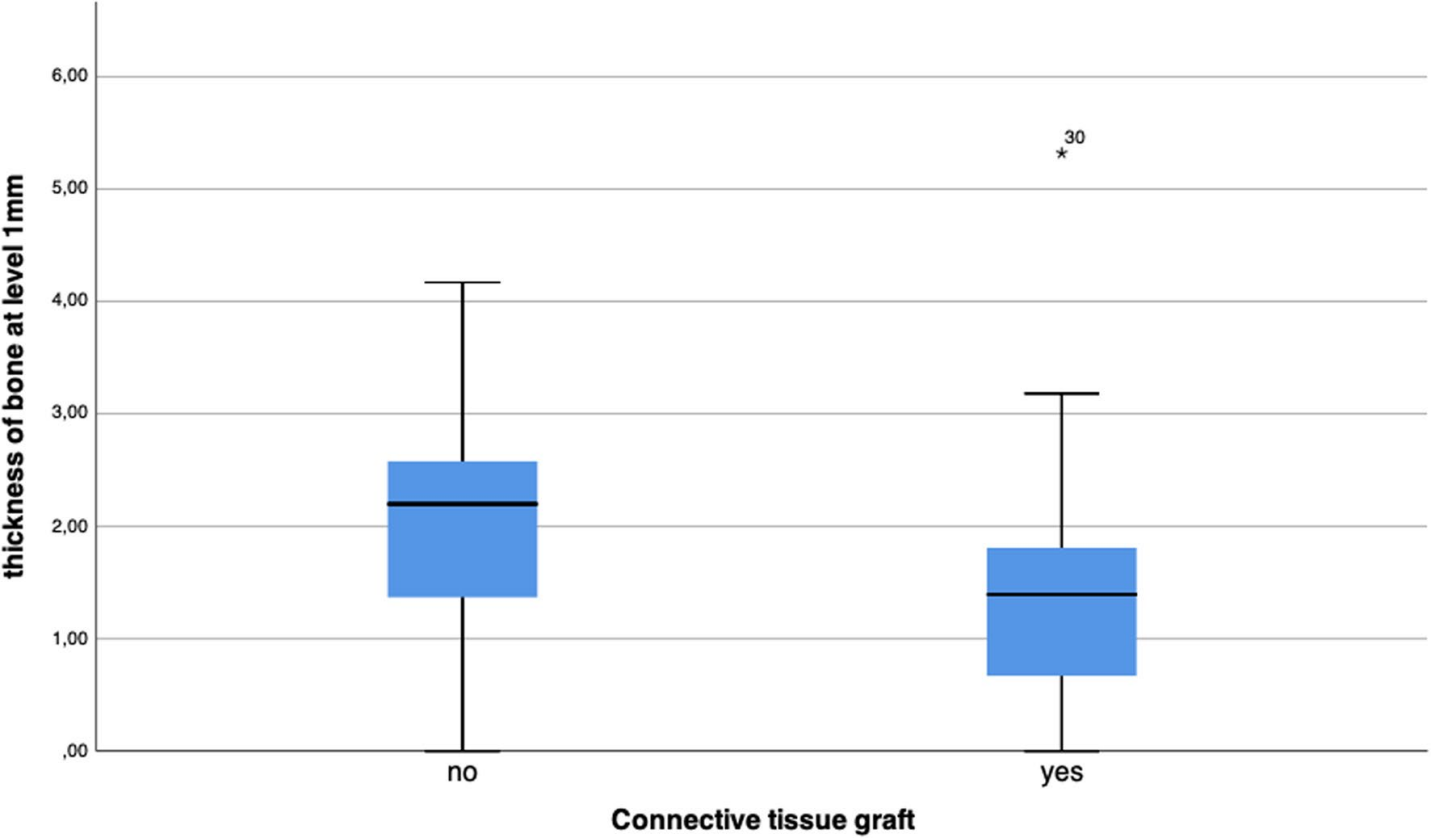
The buccal bone wall was significantly thicker in sites without CTG compared to sites with CTG at level 1 mm below reference level (p = 0.015)

The buccal bone wall was thicker in sites without CTG compared to sites with CTG at level 1 mm (2.10 ± 0.97 mm vs. 1.44 ± 1.09 mm, p = 0.015) (Fig. 6), at level 3 mm (2.20 ± 1.24 mm vs. 1.72 ± 1.18 mm, p = 0.095), and at level 6 mm (2.18 ± 1.33 mm vs. 1.56 ± 1.04 mm, p = 0.072). Additionally, the buccal bone wall was thicker in sites with thick vs. thin gingival biotype at level 1 mm (1.84 ± 1.18 mm vs. 1.56 ± 1.00 mm; p = 0.473), at level 3 mm (2.25 ± 1.29 mm vs. 1.46 ± 0.98 mm; p = 0.023), and at level 6 mm (2.16 ± 1.25 mm vs. 1.34 ± 1.01 mm; p = 0.016).

Smoking and implant shoulder design did not have an impact on the thickness of the buccal bone wall at final examination.

### Secondary outcomes

#### Interproximal marginal bone level

The mean interproximal marginal bone level was at the level of the implant shoulder (0.00 ± 0.53 mm; range, from − 1.91 to 1.38 mm) at the final examination. In sites augmented additionally with a CTG the interproximal marginal bone level was significantly lower (− 0.16 ± 0.54 mm) than in sites grafted just with AB (0.23 ± 0.48 mm) (p = 0.005). Neither smoking (p = 0.171), gingival biotype (p = 0.231) nor implant shoulder design (p = 0.103) had an impact on the interproximal marginal bone stability.

#### Soft tissue recession

**Fig. 7:**
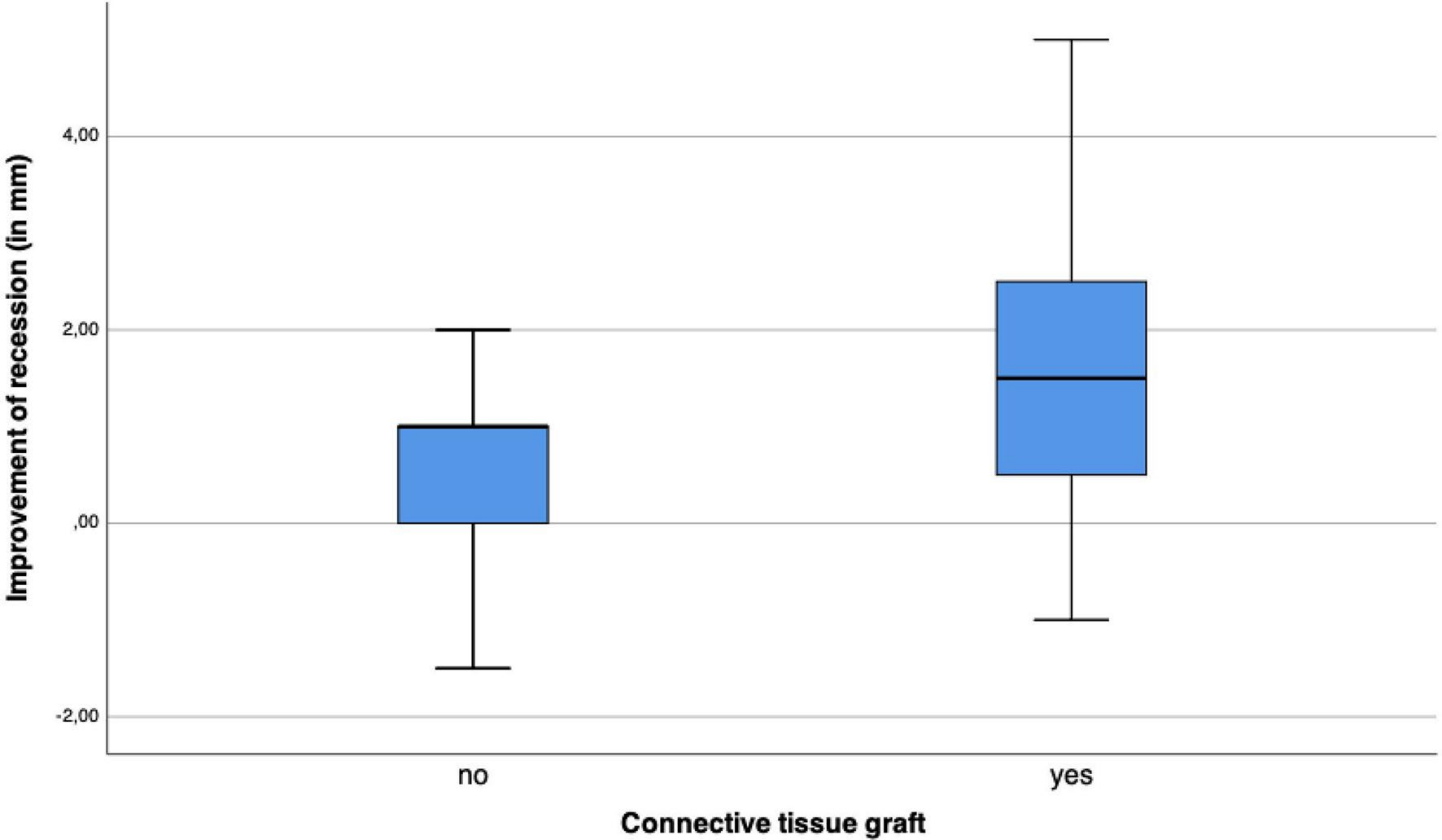
In sites with CTG the mean recession improved significantly more compared to sites without CTG (p = 0.016)

The mean initial gingival recession was 2.17 ± 1.79 mm (range, 0 to 7 mm) and improved to a mucosal recession of 0.94 ± 0.88 mm (range, 0 to 3 mm) at implant site at the final examination. The mean recession improved significantly within the treatment by 1.21 ± 1.39 mm (range, − 1.50 to 5 mm) (p < 0.001). In sites with CTG the mean recession improved significantly more (1.64 ± 1.54 mm) compared to sites without CTG (0.61 ± 0.88 mm) (p = 0.016) (Fig. 7). The gingival biotype (p = 0.93) as well as smoking status (p = 0.884) did not influence the amount of soft tissue regeneration significantly.

#### Peri-implant soft tissue esthetics

**Fig. 8:**
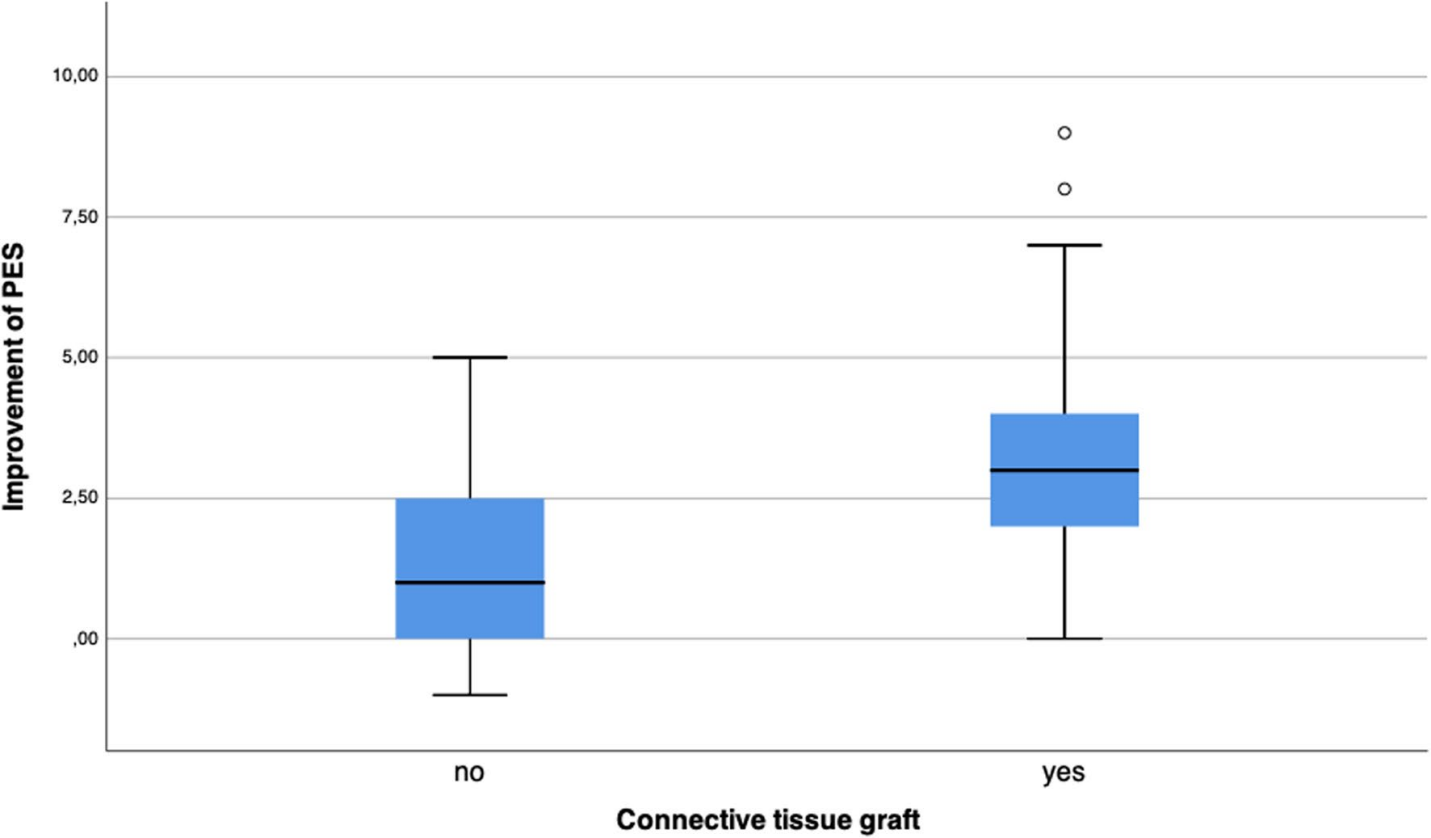
In sites with a CTG the mean PES improved significantly more compared to sites without CTG (p = 0.003)

The PES improved significantly from pre-operative to the final examination from 8.66 to 11.28 (p < 0.001). In sites with a CTG the mean PES improved significantly more (3.30 ± 2.11) compared to sites without CTG (1.50 ± 1.67) (p = 0.003) (Fig. 8). Furthermore, smoking (p = 0.847), implant shoulder design (p = 0.252) as well as gingival biotype (p = 0.795) did not influence the improvement of the PES significantly.

#### Peri-implant probing depths

The mean PPD at the final examination ranged between 1.33 and 4.17 mm at the buccal, and between 1.67 and 4.33 mm at the lingual aspect. For details see Table 1.

**Table 1:** Peri-implant probing depths at final examination

	Minimum	Maximum	Mean	SD
Mesiobuccal	2.00	5.00	2.74	0.78
Buccal	1.00	5.00	1.93	0.88
Distobuccal	1.00	5.00	2.59	0.76
Mesiooral	1.00	5.00	2.85	0.88
Oral	1.00	4.00	2.21	0.67
Distooral	1.00	5.00	2.74	0.89

#### Width of keratinized mucosa

The mean KMW at the final examination was 4.12 ± 1.81 mm (range, 0.5 to 9 mm). The KMW was not influenced by additional CTG (without CTG 4.29 ± 2.14 mm, with CTG 3.99 ± 1.54 mm; p = 0.529).

#### Correlation results

**Fig. 9:**
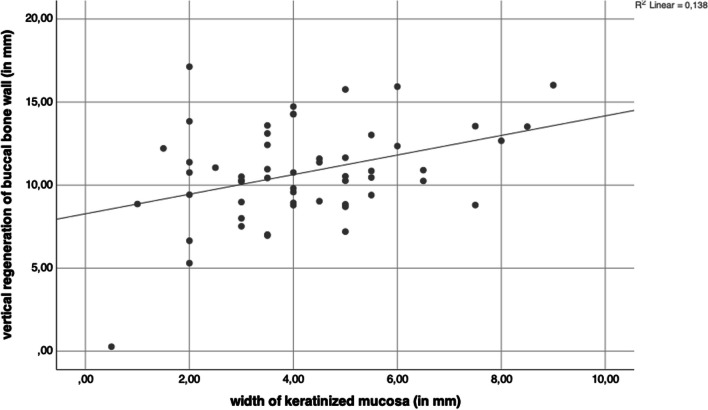
The KMW at final examination had a significant impact on the vertical buccal bone wall regeneration (r = 0.268; p = 0.048)

**Fig. 10:**
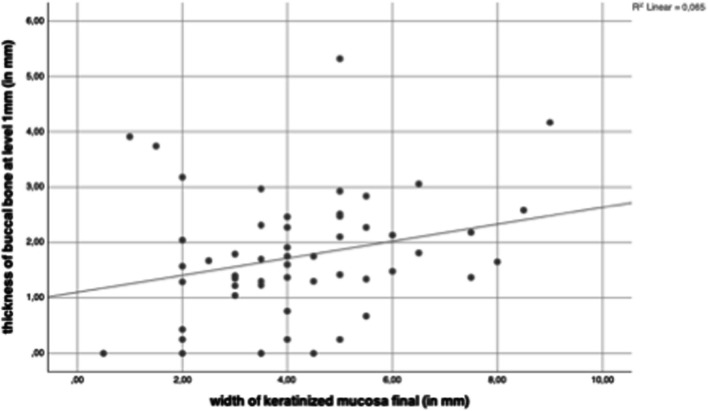
The KMW at final examination had a significant impact on the thickness of the buccal bone wall at level 1 mm below reference level (r = 0.281; p = 0.037)

The KMW at the final examination had a significant impact on the vertical buccal bone wall regeneration (r = 0.268; p = 0.048) (Fig. 9) and on the thickness of the buccal bone wall at level 1 mm (r = 0.281; p = 0.037) (Fig. 10). Even the results reached the level of significance the correlation coefficient was in the weak range.

## Discussion

This study evaluated primarily the impact of a CTG on the buccal bone regeneration in cases with a total loss of the buccal bone wall treated by immediate and flapless implant insertion and reconstruction with AB. To the best of our knowledge there is still a lack of literature about this important topic.

According to a previous study, IIP and guided bone regeneration with membranes and bone substitutes in sites with severe facial bone defects led to soft tissue deficiencies and gingival recessions, regardless of immediate or delayed provisionalization [27]. Connective tissue collapses and bone resorption due to inflammatory reactions on allo- or xenografts should be avoided in critical esthetic situations. IIP with AB in combination with a flapless approach can support the marginal soft tissue contour and the peri-implant bone regeneration [33]. Our long-term results reveal that this treatment strategy is predictable and the bone regeneration successful.

In daily practice every clinician has to deal with severe initial buccal bone deficiencies following to vertical root fractures, periapical infections or traumata but it nevertheless still represents a contraindication for IIP [5] and most often a delayed approach for implant placement is recommended [26]. In contrast to these recommendations after a mean follow-up period of 5 years, no implant was lost in our study cohort and the majority achieved and maintained a good and satisfying hard and soft tissue integration.

Consistent with our findings, Slagter et al. [47] also reported in their recent randomized clinical trial comparing immediate single-tooth implants in sockets with buccal bone defects ≥ 5 mm with a delayed approach followed by an alveolar ridge preservation, a 100% survival rate at 5-year evaluation. At the 5-year evaluation they couldn’t find any significant difference between both treatment procedures. It is noted that this study used the immediate protocol in a two step surgical procedure with submerged healing and delayed provisionalisation after 3 months compared to our single step approach with immediate provisionalisation. This small difference in the surgical protocol may be an explanation for the slightly better interproximal bone levels in our study results.

We note additionally that while our finding of the reconstruction of the buccal bone wall is consistent with the results of Slagter et al. [47], the buccal bone wall in the presented study was thicker at final examination even though the initial buccal bone defect was more severe. This leads to the hypothesis that the bigger the initial bone defect the greater the potential for bone regeneration, which was reported in an earlier publication already [32, 34, 35].

In over 90% of the cases a full reconstruction of the buccal bony wall up to the neck of the implant and above was observed. One implant had CB-CT data showing no buccal bone at all. It was rated as a reconstruction failure but this result does not mean that buccal bone wall is completely missing because the buccal bone thickness has to be at least 0.5 mm to be detected on CB-CT images [21].

In the study of da Rosa et al. the reconstruction of severe buccal bone wall defects was observed in 18 cases with a follo-w-up of 58 months. The successful reconstruction of the buccal bone wall was radiographically presented in 2 cases but not statistically analyzed in dimension in their study group [42].

In our study from 2011 CBCTs were available for 16 patients after a mean follow-up of 36 months; in 12 cases a full bone regeneration and in 4 cases a partial regeneration was documented without analyzing the dimension of the bone wall [33]. Covani et al. [14] found a full bone reconstruction in 70% of their cases with total loss of the buccal bone wall with and without flap elevation, as well as a more favorable vertical bone regeneration at the buccal aspect in sites with flap elevation. In the subsequent years flapless surgery established itself as a very successful treatment option [33, 42, 46] and the latest results of our current study confirm that a buccal bone defect regeneration can be achieved with a flapless approach.

The principal risk factors of IIP and immediate peri-implant bone reconstruction are facial soft tissue recessions and orofacial flattening of the soft tissue profile [13, 26]. Furthermore, severe resorption of the buccal bone wall from 36% [3] to 57% [31] according to the reported CB-CT data have been observed. In contrast to the aforementioned risks, we could find a mean improvement in soft tissue recession by 1.2 mm after a mean observation period of 5 years which shows sustainability of the final results.

Consistent with our findings, van Nimwegen et al. [51] also reported increased mid-facial vertical soft tissue levels after using IIP in combination with a CTG [51]. But they also noted that the resorption of the facial bone wall cannot be compensated completely by the use of a CTG. In contrast, Fujita et al. [22] showed that the soft tissue gain after IIP with CTG can compensate the bone resorption and preserve the preoperative mucosal contour [22].

A recently published systematic review affirmed the positive effect of simultaneous soft tissue augmentation on marginal bone levels (De Angelis [15]). This is in line with the results of an earlier study of our group discussing the successful outcomes after an IIP in sites with initial recessions grafted with autogenous bone. In the subgroup with an additional CTG we found more vertical bone regeneration, a thicker buccal bone wall, a more coronal buccal bone level and less bone resorption in the follow-up [32, 34, 35]. In contrast, we found in the present study that in cases with a total loss of the buccal bone an additional CTG had a significant negative impact on the marginal bone level and buccal bone thickness.

These results were unexpected, but we assume that the two different tissues compete against each other for the existing space in between implant surface and the remaining facial tissues. In cases with a total loss of the buccal bone there might be no periosteum left on the buccal defect side as well. Additionally, the CTG might increase the pressure on the grafted bone in the early healing period.

A recently published randomized clinical study from Zuiderveld et al. [56] also stated a decrease in buccal bone thickness when a CTG was used in a single immediate implant site although they only include cases with a pristine buccal bone wall [56]. They also assumed that the disruption of the blood supply in combination with the bone remodeling process after tooth extraction could have induced the increased loss of buccal bone wall compared to sites without CTG. In contrast to our surgical approach using a CTG fixed subperiosteally and extended to the adjacent teeth, Zuiderveld et al. used smaller CTG with a length of 8 mm, which were placed in a supraperiostal envelope flap covering just the implant site.

Another important secondary outcome variable of the present study was the evolution of the peri-implant soft tissue esthetics evaluated by the PES. The mean PES improved from 8.66 pre-operative to 11.28 at the final examination. The PES improvement in sites with CTG was twice as much as in sites without simultaneous soft tissue grafting. This supports the aforementioned conclusion that the use of a CTG can optimize the esthetic outcome after IIP and the results are consistent with earlier study data of Noelken et al. [32, 32, 34, 34, 35, 35]. The study of Pohl evaluated the esthetic changes after IIP and immediate provisionalization in sites with severe buccal bone deficiencies [39]. They found no statistical significant improvement of the PES when grafted with bovine bone collagen and unchanged esthetics when sites were not grafted at all. They did not use an additional CTG. Finally, they concluded that the treatment concept even without a facial bone wall is successful, but the patient has to accept the initial esthetic situation since their treatment strategy was not able to change this predictably.

The limitation of our inclusion criteria to Astra Tech implants, a flapless procedure, and autogenous bone chips reduces the external generalizability of the results but also minimizes the heterogeneity of the treatment concept in this cohort. By this we were able to document a reliable treatment option for cases with a total loss of the buccal bone wall which was successful in the long run. Furthermore, all surgical procedures were performed by the same oral surgeon with experience in implant surgery over 30 years to eliminate the surgeon bias, but this leads to the assumption that this technique is quite sensitive and should only be performed by experienced clinicians.

As a limitation of this surgical approach, we need to address that the scientific evidence for IIP in cases with severe recessions is very limited [30, 48]. Severely compromised alveolar sockets with massive periradicular infection leading to insufficient primary stability represent another contraindication.

Prospective comparative studies from independent groups are encouraged to clarify the advantage of using autogenous bone chips versus other protocols for guided bone regeneration of buccal bone deficiencies, as they will serve to externally validate the reported results.

Keeping in mind that scientific research data on IIP and immediate reconstruction by using the flapless technique and autogenous bone in combination with a CTG in cases of total loss of the buccal bone wall are still limited and mostly observational, this approach represents an advanced treatment option with the potential of a high success rate in the hands of a skilled oral surgeon. Furthermore, it seems to offer promising mid to long-term marginal bone stability and favourable esthetic outcomes.

## Conclusion

Within all the limitations the radiographic and clinical results of this retrospective study, proof of principle has been provided that implant insertion into fresh extraction sockets can be successfully performed without flap elevation even in the presence of total buccal bone loss. Buccal defect grafting with autogenous bone chips led to a favourable vertical buccal bone wall reconstruction. An additional connective tissue graft reduced the gingival recession and improved the soft tissue esthetics but also reduced the thickness of the buccal bone wall as well.

## Data Availability

The dataset used and analysed during the current publication is available from the corresponding author on reasonable request.

## Abbreviations

AB: Autogenous bone
BBT: Buccal bone thickness
CB-CT: Cone beam computed tomography
CEJ: Cementoenamel junction
CTG: Connective tissue grafting
IIP: Immediate implant placement
KM: Keratinized mucosa
KMW: Width of keratinzed mucosa
PES: Pink Esthetic Score
PPD: Peri-implant probing depths
